# A Sulfated Polysaccharide from Red Seaweed *Gracilaria caudata* Exhibits Antioxidant and Antiadipogenic Activities In Vitro

**DOI:** 10.3390/md24010015

**Published:** 2025-12-26

**Authors:** Maxsuell Lucas Mendes Marques, Leandro Silva Costa, Mariana Santana Santos Pereira Costa, Hugo Alexandre Oliveira Rocha

**Affiliations:** 1 Graduate Program in Health Sciences, Federal University of Rio Grande do Norte—UFRN, Natal 59078-970, Rio Grande do Norte, Brazil; maxsuell_lucas@hotmail.com; 2 Federal Institute of Education, Science, and Technology of Rio Grande do Norte (IFRN), Canguaretama 59190-000, Rio Grande do Norte, Brazil; leandro-silva-costa@hotmail.com; 3 Federal Institute of Education, Science, and Technology of Rio Grande do Norte (IFRN), João Câmara 59550-000, Rio Grande do Norte, Brazil; mariana.costa@ifrn.edu.br; 4 Natural Polymer Biotechnology Laboratory (BIOPOL), Department of Biochemistry, Center of Biosciences, Federal University of Rio Grande do Norte—UFRN, Natal 5908-970, Rio Grande do Norte, Brazil

**Keywords:** sulfated polysaccharide, oxidative stress, 3T3-L1 cells, anti-obesity, sulfated galactan

## Abstract

This study investigated the antioxidant and antiadipogenic activities of sulfated polysaccharide (SPs) from the red seaweed *Gracilaria caudata*. First, sulfated polysaccharide-rich extracts (SPREs) from fifteen tropical seaweeds were screened to evaluate both their chemical composition and antioxidant potential. Among all samples, *G. caudata* exhibited the highest total antioxidant capacity, which justified its selection for detailed characterization. Sequential acetone precipitation produced three SPs (F1.5, F2.0, and F3.0), differing in sulfate content, monosaccharide composition, and molecular weight. In vitro assays revealed that F1.5 had the highest total antioxidant capacity and strong iron-chelating activity, while F2.0 exhibited the most effective hydroxyl radical scavenger. Importantly, F1.5 was the only SP that was non-cytotoxic to non-tumor cell lines. In 3T3-L1 preadipocytes, F1.5 attenuated H_2_O_2_-induced oxidative stress by reducing ROS and MDA levels and restoring GSH and SOD activity, achieving effects comparable to those of quercetin. Moreover, F1.5 inhibited adipogenic differentiation in a dose-dependent manner, as evidenced by decreased Oil Red O staining and reduced glycerol release. Collectively, these findings indicate that F1.5 exerts both antioxidant and antiadipogenic activities, highlighting *G. caudata* as a promising natural source of bioactive polysaccharides with potential nutraceutical applications. Nonetheless, further studies are required to elucidate the molecular mechanisms underlying these effects, validate the efficacy in vivo, and assess bioavailability and safety before clinical translation can be considered.

## 1. Introduction

Obesity is a condition characterized by the abnormal or excessive accumulation of adipose tissue in the body, and it is currently recognized as one of the most serious public health challenges on a global scale [1]. Beyond its high prevalence, obesity is compounded by its frequent association with various metabolic and inflammatory comorbidities, including type 2 diabetes mellitus [2], hypertension [3], dyslipidemia [4], cardiovascular diseases [5], and different types of cancer [6].

The epidemiological monitoring of obesity is primarily conducted using the Body Mass Index (BMI), expressed in kg/m^2^. Based on this parameter, individuals with a BMI equal to or greater than 30 kg/m^2^ are classified as obese. Under this criterion, an alarming growth in obesity has been observed in recent decades [7]. In fact, the World Health Organization [1] reported that its prevalence has more than doubled over the past thirty years, with data from 2016 indicating approximately 650 million obese adults worldwide.

A notable physiopathological aspect of obesity is the increase in oxidative stress, characterized by an imbalance between the production of reactive oxygen species and endogenous antioxidant mechanisms. This condition has led researchers to suggest that oxidative stress may not only result from obesity but also contribute to its development and progression [8]. In this context, compounds with antioxidant activity are emerging as promising agents in the prevention and management of obesity-associated complications [9].

There is currently increasing interest in substituting synthetic antioxidants with natural compounds, due to the safety, biocompatibility, and potential multifunctionality of the latter. This scenario has driven studies focused on prospecting natural sources, chemical characterization of raw materials, and identifying new bioactive antioxidants [10]. In therapeutic terms, the use of natural products with antioxidant potential represents an appealing alternative for obesity management, considering that many available pharmaceuticals present adverse effects and high costs [11].

Marine algae, in turn, have been used as food and a medicinal resource since antiquity by different cultures globally [12]. These organisms synthesize a wide array of bioactive compounds, including pigments, polyphenols, and polysaccharides, many of which exhibit significant antioxidant activity [13]. Among these compounds, sulfated polysaccharides (SPs) are prominent, known for their antioxidant properties and their protective role against oxidative stress [14].

As part of our efforts to identify marine sources of bioactive sulfated polysaccharides, we extracted and evaluated sulfated polysaccharide-rich extracts (SPRE) from fifteen species of tropical seaweeds collected along the Northeastern coast of Brazil (see topic 2.1). The chemical and antioxidant screening revealed distinct patterns among the extracts, with those derived from red algae showing particularly high activity. Notably, the SPRE obtained from the edible *Gracilaria caudata* exhibited the strongest total antioxidant capacity (TAC) among all samples tested, surpassing other red, brown, and green algae. This finding confirmed *G. caudata* as a promising source of antioxidant sulfated polysaccharides and provided the rationale for its selection for further isolation, structural characterization, and functional evaluation.

Given the strong relationship between oxidative stress and obesity, the search for natural compounds with antioxidant and lipid metabolism modulatory potential has become a promising strategy for developing complementary and preventive therapies. In this context, the SPs from *G. caudata* emerge as notable candidates.

The alga *G. caudata* is recognized for its wide occurrence on the Brazilian Northeast coast and its potential to produce high-yield sulfated polysaccharides with structural complexity. Nevertheless, there are still limited studies exploring its potential antioxidant actions [15], and no studies on its antiadipogenic effects exist. Therefore, the investigation of *G. caudata* SPs represents an opportunity to contribute not only to the advancement of knowledge regarding marine polysaccharides but also to the development of natural compounds with potential application in the prevention and control of obesity and its associated comorbidities.

## 2. Results and Discussion

The first step of this study consisted of obtaining and screening sulfated polysaccharide-rich extracts (SPRE) from fifteen tropical seaweed species to identify potential sources of natural antioxidants. The results of this comparative analysis, which are summarized in Table 1, serve as the experimental basis for the observations discussed in the Introduction and guided the subsequent selection of *G. caudata* for detailed investigation.

### 2.1. Chemical Analysis and Antioxidant Activity of Sulfated Polysaccharide-Rich Extracts

Using a combined methodology of proteolysis and acetone precipitation, we obtained sulfated polysaccharide-rich extracts (SPRE) from fifteen tropical seaweeds collected along the Northeastern coast of Brazil. The chemical composition of the SPRE is summarized in Table 1. Among the species analyzed, the red alga *G. caudata* exhibited the lowest sugar-to-sulfate ratio (0.19). In brown algae (Phaeophyta), the sugar-to-sulfate ratio ranged from 0.20 in *D. menstruallis* to 0.68 in *D. justii*. Therefore, except for *C. cupresoides*, all green algae (*Chlorophyta*) yielded SPRE with high sugar-to-sulfate ratio, particularly *C. prolifera* (1.19), *C. sertularioides* (0.80), *Udotea flabellum* (0.76) and *C. isthmocladum* (0.65).

Regarding antioxidant activity (Table 1), the SPREs exhibited distinct performances, despite all containing sulfated polysaccharides in their composition. Among the green algae, the SPRE from *C. isthmocladum* showed the lowest antioxidant efficiency, whereas the SPRE from *U. flabellum* displayed significantly higher activity. Most of the SPREs derived from brown algae demonstrated comparable antioxidant capacity; however, extracts from *S. filipendula* and *D. justii* stood out by exhibiting significantly stronger activity than the other brown algal SPREs. Notably, the most efficient antioxidant extract overall was obtained from the red alga *G. caudata*, which presented significantly greater activity than all other SPREs evaluated.

The Total Antioxidant Capacity (TAC) assay was chosen because it is conducted in the presence of sulfuric acid and under elevated temperature, conditions that promote the transfer of all available electrons from the sample to the oxidant, in this case, molybdate (Mo^6+^).

This methodological choice is particularly useful when one wants to compare antioxidant potentials across multiple extracts, since in theory it accounts for the full electron-donating capability of the antioxidants present. In our study, however, no correlation was observed between TAC values and the sugar/sulfate ratio of the polysaccharide extracts, suggesting that structural features beyond simple sulfate content, such as monosaccharide type, sulfation site, molecular weight, and three-dimensional conformation, play a major role in determining antioxidant behavior as described for other sulfated polysaccharides [16].

Since the SPRE from *G. caudata* exhibited the highest antioxidant activity among all extracts, it was selected for further purification to isolate and characterize its constituent sulfated polysaccharides. This decision was based on the hypothesis that the remarkable antioxidant performance of this extract is primarily attributable to its sulfated polysaccharides. Supporting this, Alencar et al. [17] demonstrated that sulfated agaran from *G. caudata* display antioxidant activity in vitro, as evidenced by their total antioxidant capacity and ferrous ion-chelating ability. These findings reinforce the view that sulfated polysaccharides are major contributors to the antioxidant potential of *G. caudata*.

### 2.2. Isolation and Chemical Characterization of Sulfated Polysaccharides from G. caudata

We used a low-cost, widely reproducible method, which combined proteolysis and sequential acetone precipitation, to obtain three sulfated polysaccharides (named F1.5; F2.0; and F3.0) from the *G. caudata*. The results of the chemical analysis of the fractions and their polysaccharide yields are summarized in Table 2. Phenolic compounds were not detected in any of the samples. In addition, protein was only present at trace levels in F1.5 and F2.0 and was not detected in F3.0. Conversely, the combined carbohydrate and sulfate contents accounted for nearly 100% of the material, indicating that the samples are predominantly composed of sulfated polysaccharides.

Chemical analyses revealed that F2.0 exhibited the highest sulfate content. In addition, F2.0 and F3.0 contained galactose, mannose, glucose, and xylose, but in different proportions, highlighting the structural heterogeneity of *G. caudata* polysaccharides. Such heterogeneity is noteworthy when compared to the relatively homogeneous sulfated galactans commonly reported in *Gracilaria millardetii* and *Gracilaria textorii* [18] and *Gracilaria intermedia* [19]. Nevertheless, similar complexity has been described for polysaccharides from *Gracilaria chouae* [20] and *Gracilaria fisheri* [21], in which galactose backbones are substituted with xylose and glucose residues.

**Figure 1:**
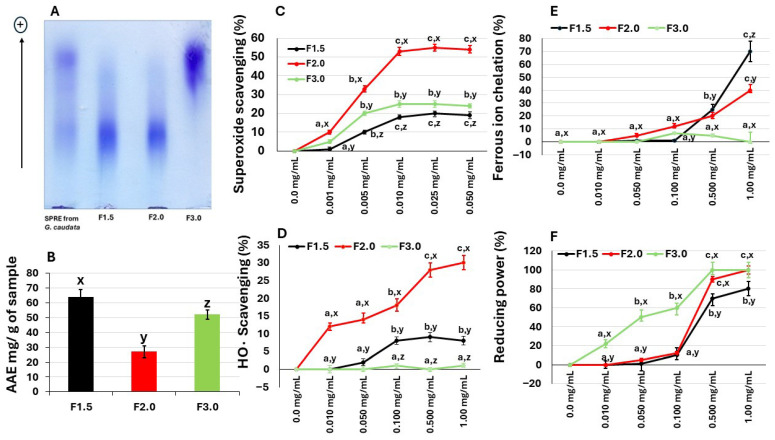
(**A**) Electrophoretic analysis of sulfated polysaccharides from *G. caudata*, performed in 0.05 M diaminopropane acetate buffer (pH 9.0). Approximately 5 µL (50 µg) of each polysaccharide fraction was loaded onto an agarose gel prepared with the same buffer and subjected to electrophoresis as described in the Methods Section. (+) Positive pole. (**B**) Total antioxidant capacity of the samples. (**C**) Superoxide radical scavenging activity. (**D**) Hydroxyl radical scavenging activity. (**E**) Iron-chelating activity. (**F**) Reducing the power of the samples. Data are expressed as mean ± standard deviation from three independent experiments. Different lowercase letters (a, b, c) indicate significant differences (*p* < 0.01) among concentrations of the same polysaccharide. Different lowercase letters (x, y, z) indicate significant differences (*p* < 0.01) among polysaccharides from *G. caudata* at the same concentration.

To confirm whether sulfate was covalently linked to polysaccharides, we subjected the fractions to agarose gel electrophoresis. After the gel was stained with toluidine blue (Figure 1) we found that all fractions contained electrophoretically mobile, purple-colored bands, characteristic of sulfated polysaccharides.

The extraction method employed, combining proteolysis and sequential acetone precipitation, yielded three distinct sulfated polysaccharide fractions (F1.5, F2.0, and F3.0). The absence of phenolic compounds in all fractions confirmed that the bioactivity observed in subsequent assays could be attributed mainly to the polysaccharides.

Electrophoretic analysis using toluidine blue confirmed the sulfated nature of these molecules, as evidenced by the presence of purple bands, a characteristic feature of sulfated polysaccharides. Fractions F1.5 and F2.0 displayed similar electrophoretic mobility, suggesting that they belong to the same structural family, whereas F3.0 showed a distinct migration pattern, indicating structural differences. This result is consistent with previous studies reporting that marine macroalgae contain more than one population of sulfated polysaccharides with variable electrophoretic behavior [22,23].

Despite the shared electrophoretic profile of F1.5 and F2.0 and molecular weight, their distinct chemical compositions suggest subtle structural differences that may translate into different biological activities. Comparable findings were reported for sulfated polysaccharides from *C. isthmocladum*, where SPs with similar electrophoretic mobility presented distinct monosaccharide compositions and sulfate contents [23].

A high-molecular-weight sulfated polysaccharide (≈250 kDa), classified as an agaran, was purified from *G. caudata* in 2013 [24]. Its structure was characterized by partial substitution of hydroxyl groups with methyl and pyruvate acetal moieties and by a very low degree of sulfation (≈1%). Sulfate groups were specifically located at the C-6 position of L-galactose residues, which are the biological precursors of 3,6-anhydro-L-galactose. These characteristics define a nearly neutral agaran with high molecular homogeneity.

Subsequently, in 2019, another agaran was isolated and characterized from the same alga [15]. This polysaccharide displayed a considerably lower molecular mass (≈116 kDa) and a markedly higher sulfate content (≈8.5%), indicating a more heterogeneous and anionic structure. Importantly, this agaran exhibited antioxidant activity both in vitro and in vivo, suggesting that its biological functionality is closely related to the degree and distribution of sulfation along the galactan chain.

Taken together, these findings demonstrate that *G. caudata* can produce structurally distinct agarans that differ in molecular mass and sulfation pattern. Based on these parameters, the F1.5 fraction obtained in the present study corresponds to the polysaccharide described by Alencar et al. [15], whereas fractions F2.0 and F3.0 represent previously unreported agarans. In this context, these three polysaccharides were subsequently subjected to comprehensive evaluation of their antioxidant potential in vitro and in cell-based assays.

### 2.3. In Vitro Antioxidant Activity of Sulfated Polysaccharides from G. caudata

It is generally acknowledged that no single antioxidant assay can accurately reflect a compound’s activity across all biological conditions. Therefore, a comprehensive evaluation is essential to fully characterize a compound’s antioxidant potential. In this study, the sulfated polysaccharides from *G. caudata* were subjected to five distinct in vitro antioxidant assays, targeting diverse inherent properties crucial for an effective antioxidant agent: ferric ion chelation, various forms of radical scavenging capacity, and reducing power.

The initial assay quantified the TAC of the fractions, as illustrated in Figure 1B. F1.5 exhibited the highest antioxidant activity, demonstrating an equivalent capacity of 63.9 mg ascorbic acid per gram of fraction (mg AA/g). This value was statistically significantly higher (*p* < 0.01) than those recorded for F2.0 (25.4 mg AA/g) and F3.0 (53.0 mg AA/g), respectively. F1.5 also presented a TAC value higher than that of the SPRE from *G. caudata* (55.8 mg AA/g—Table 1). These results show that the SPS from the algae *G. caudata* have antioxidant potential, which led to the performance of other antioxidant assays.

Superoxide anion (O_2_^∙−•^) is a key reactive oxygen species (ROS) in biological systems, essential for oxygen consumption during ATP synthesis and storage within mitochondria [25]. However, excessive levels of superoxide anion contribute to oxidative stress and, consequently, to the development and progression of various diseases [26]. Therefore, the F1.5, F2.0, and F3.0 samples were evaluated for their ability to scavenge superoxide anions.

As shown in Figure 1C, all three samples demonstrated a scavenging activity that stabilized at approximately 0.01 mg/mL and did not increase further even with elevated concentrations. Notably, samples F1.5 and F3.0 exhibited modest activity, reaching a maximum of 20% inhibition. Despite this limited efficacy, their activity was superior to that of the agaran (sulfated homogalactan) from *G. birdiae*, which showed no superoxide anion scavenging activity even at high concentrations (0.5 to 2.0 mg/mL) [27]. In contrast, sample F2.0 displayed significantly higher activity, reaching over 50% inhibition.

The neutralization of superoxide anions is hypothesized to occur through mechanisms involving electrostatic interactions or electron donation, where the presence of sulfate groups is considered a major contributing factor [28,29]. The higher sulfate content detected in F2.0, relative to F1.5 and F3.0, may justify its superior activity in this assay. Nevertheless, further studies are required to confirm this potential correlation.

The polysaccharides from *G. caudata* were evaluated for their ability to scavenge the hydroxyl radical (•OH), which is considered the most reactive and cytotoxic among the free radicals generated in biological systems. Among the tested fractions, F2.0 exhibited the most pronounced activity, achieving approximately 30% inhibition at 1.0 mg/mL, whereas F1.5 and F3.0 displayed only residual or negligible scavenging effects (Figure 1D).

The comparatively low activity of fractions F3.0 and F1.5 may be associated with structural variations, particularly in sulfation degree and molecular size. Previous studies on ulvans [30] and carrageenans [31] have shown that antioxidant capacity, especially hydroxyl radical scavenging, increases with higher sulfation levels. Therefore, the superior activity observed for the F2.0 fraction may result from an optimal balance between sulfation density and chain length, enhancing its reactivity toward hydroxyl radicals.

Metal chelation assays are commonly employed to evaluate the in vitro antioxidant activity of various substances. Accordingly, F1.5, F2.0 and F3.0 samples were tested for their capacity to chelate iron (Figure 1E).

Iron chelators play a protective role against oxidative stress by removing Fe^2+^ ions, which are known to participate in hydroxyl radical generation via Fenton reactions. However, under the conditions tested, F3.0 samples did not exhibit measurable iron-chelating activity. This lack of activity has also been reported for other sulfated polysaccharides, commercial fucoidans derived from brown seaweeds *Undaria pinnatifida*, *Macrocystis pyrifera*, and *Fucus vesiculosus* [32].

In contrast, the F2.0 and F1.5 samples demonstrated the ability to chelate iron ions, as shown in Figure 1E. The effect was dose dependent. At a concentration of 0.1 mg/mL, F2.0 exhibited an iron chelating capacity of 5.3%, while F1.5 showed 10%. At 1.0 mg/mL, the chelating capacities increased to 40% for F2.0 and 70% for F1.5.

Excessive iron accumulation in the body, arising from genetic disorders such as hereditary hemochromatosis, repeated blood transfusions, or chronic liver disease, leads to the generation of reactive oxygen species through Fenton and Haber–Weiss reactions, thereby inducing oxidative stress, lipid peroxidation, and tissue damage in organs such as the liver, heart, and pancreas [33]. Current therapeutic approaches focus on reducing systemic iron burden via chelating agent, such as deferoxamine, deferiprone, and deferasirox, which promote iron mobilization and excretion through urine or feces. Although these agents have demonstrated clinical efficacy, their use is frequently associated with adverse effects and limited tissue selectivity, highlighting the continuing need for safer and more effective iron chelators with improved pharmacokinetic and pharmacodynamic properties [34].

In this context, the results of the present study indicate that F1.5 may represent a promising iron-chelating candidate for future in vivo evaluation. Moreover, given its demonstrated metal-binding capacity, F1.5 can be classified as a preventive antioxidant, capable of mitigating oxidative damage through the sequestration of transition metal ions.

The reductive activity profile observed for *G. caudata* polysaccharides indicates that all samples possess the ability to donate electrons, thereby acting as reducing agents within the Fe^3+^ → Fe^2+^ system. This finding aligns with previous studies that reported a dose-dependent reducing effect for sulfated polysaccharides derived from red and brown algae [35]. The higher efficiency exhibited by the F3.0 fraction at lower concentrations (0.01 to 0.1 mg/mL) suggests that its structural composition, possibly characterized by a lower molecular weight or a higher density of sulfated groups, enhances electron transfer under low-concentration conditions. Similar relationships between the degree of sulfation or molecular mass and reductive activity have been demonstrated [36]. Conversely, the observation that the activities of the fractions become more comparable at higher concentrations may be attributed to the saturation of reducing sites or to steric hindrance effects. When many molecules are present, structural differences become less influential, resulting in a convergence of overall electron-donating capacity among the extracts. This behavior has also been reported in reducing power assays of sulfated polysaccharides, where increasing concentration tends to diminish the disparity in activity between distinct fractions [37].

### 2.4. Evaluation of the Cytotoxic Effect of G. caudata Polysaccharides in Different Cells

**Figure 2:**
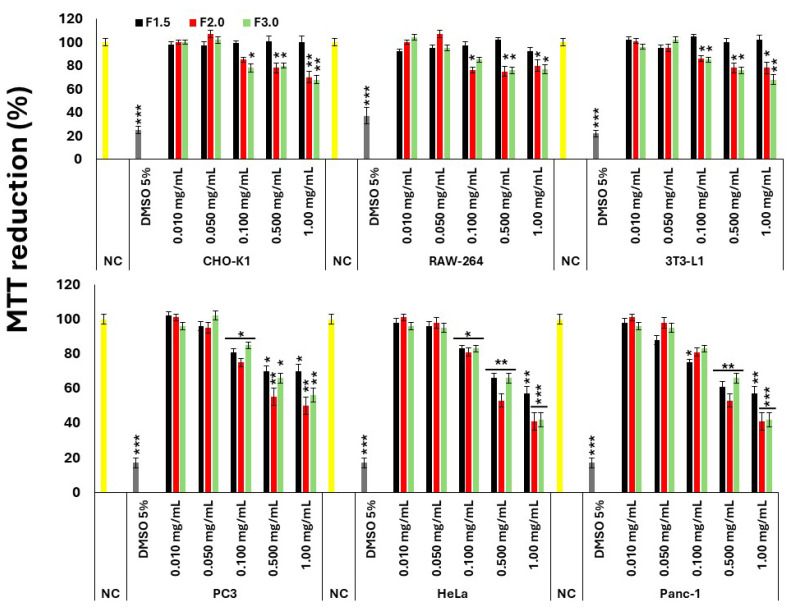
Evaluation of the cytotoxic effect of *G. caudata* polysaccharides on different cell lines NC = negative control (cells treated with DMEM + FBS only). A 5% DMSO solution was used as the positive control. The values are presented as the mean ± standard deviation (SD) from three independent experiments. * *p* < 0.05, ** *p* < 0.01, and *** *p* < 0.001 indicate significant differences compared with the negative control.

The subsequent phase of the investigation involved assessing the antioxidant activity of polysaccharides isolated from *G. caudata* within a cellular context. To establish their safety profile and potential scope of application, the cytotoxicity of these polysaccharides was initially examined across a panel of cell lines, comprising three non-tumor models (CHO, 3T3-L1, and Raw-264) and three tumor models (PC3, HeLa, and Panc-1). The corresponding results are presented in Figure 2.

All polysaccharides exhibited a dose-dependent cytotoxic effect in the three tumor cell lines evaluated. Moreover, no statistically significant differences were detected among the effects of the distinct polysaccharide fractions (F1.5, F2.0, and F3.0), except at the highest concentration tested (1.0 mg/mL). At this specific concentration, F2.0 and F3.0 demonstrated significantly greater cytotoxic activity compared to F1.5.

The scientific literature supports the potential of antioxidant polysaccharides derived from marine algae as cytotoxic agents against neoplastic cells, a topic of considerable current interest, as highlighted in recent reviews [38,39,40]. Specifically, the antitumor potential of polysaccharides from red algae (Rhodophyta) is well documented. For instance, Sae-Lao et al. [41] demonstrated that sulfated galactans isolated from *Gracilaria fisheri* inhibited the proliferation of cholangiocarcinoma cells. Similarly, polysaccharides extracted from *Gracilaria lemaneiformis* using ultrasound- and microwave-assisted extraction in an aqueous biphasic system exhibited in vitro antitumor activity by inhibiting the growth of MCF-7, HepG-2, and HeLa cell lines [42]. It should be noted, however, that determining antitumor activity was not the primary objective of the present study. Nonetheless, further investigations aimed at exploring this potential in more comprehensive in vitro and in vivo models are envisaged.

In contrast, and as also shown in Figure 2, regarding non-tumor cell lines, only F1.5 exhibited no detectable cytotoxicity at the concentrations tested. This observation is particularly relevant for potential biological applications, suggesting a favorable safety profile for F1.5 when compared to the other samples.

Moreover, the decision to prioritize F1.5 for subsequent investigations was not based solely on antioxidant performance in cell-free systems. Importantly, the assay (TAC assay) carried out at 100 °C does not aim to mimic physiological conditions, but rather to determine the full electron-donating potential of thermally stable antioxidant molecules. Under these high-temperature conditions, labile groups are denatured and weak bonds dissociate, revealing the maximum reducing capacity maintained even after thermal stress. When samples are compared by this method, the one displaying the highest result is expected to possess a more structurally robust and potentially more versatile reducing ability across cellular and extracellular microenvironments.

In this context, F1.5 exhibited the highest temperature-resistant antioxidant response, suggesting a stable and sustained electron-donating profile, an attribute commonly associated with sulfate positioning and charge density in sulfated polysaccharides. In addition to this structural robustness, F1.5 demonstrated measurable iron-chelating activity, presented the highest extraction yield (82%; Table 2) compared to F2.0 (12%), and showed no detectable cytotoxicity toward non-tumor cells at the concentrations tested. Taken together, these factors position F1.5 as the most promising and feasible fraction for continued biological and structural investigation.

### 2.5. Effect of F1.5 on H_2_O_2_-Induced Oxidative Stress

Since F1.5 was the only polysaccharide that did not exhibit cytotoxic activity toward 3T3-L1 preadipocytes, all subsequent experiments were conducted exclusively with this sample. The concentration range used in the oxidative stress assays (0.01–1.00 mg/mL) was selected based on previous studies reporting antioxidant and metabolic effects of sulfated polysaccharides from marine algae in cell models, including 3T3-L1 cells. Additionally, the initial cytotoxicity screening performed in this study demonstrated that F1.5 was non-toxic up to 1 mg/mL (Figure 2), supporting the use of this concentration as the highest safe level for subsequent assays.

**Figure 3:**
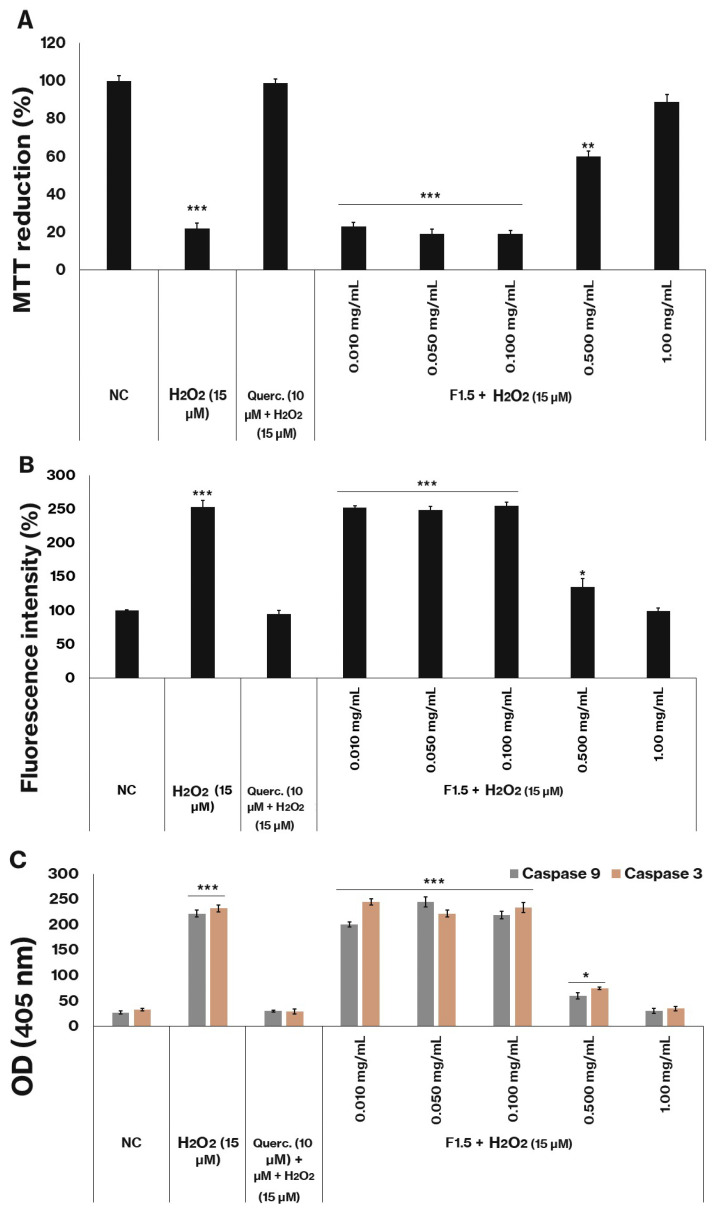
F1.5 Protects 3T3-L1 cells against H_2_O_2_-Induced Oxidative Damage by Reducing ROS production and caspase activation. (**A**)—Cell protection by F1.5 against H_2_O_2_. (**B**)—F1.5 reduces intracellular ROS levels. (**C**)—F1.5 attenuates H_2_O_2_-induced caspase-3 and caspase-9 activation. The values are presented as the mean ± standard deviation (SD) from three independent experiments. Querc. = quercetin. NC = negative control (cells treated with DMEM + FBS only). OD = optical density. * *p* < 0.05, ** *p* < 0.01 and *** *p* < 0.001 indicate significant differences compared with the negative control.

The exogenous addition of H_2_O_2_ to cells may result in three immediate outcomes: an increased propensity for the Fenton reaction, leading to the generation of hydroxyl radicals (•OH); the interaction of H_2_O_2_ with endogenous reactive molecules, thereby compromising cellular antioxidant defenses; or its rapid decomposition through catalase-mediated reduction [43]. Under conditions of excessive exposure, such as those applied in the present experiments, hydrogen peroxide elicits oxidative stress and cellular damage. This effect was evident in the positive control group of the assays, wherein the MTT reduction capacity decreased by approximately 80%, as shown in Figure 3A, indicating the cytotoxic effect of hydrogen peroxide through the induction of oxidative stress.

The data shown in Figure 3B indicates that the presence of peroxide significantly increased the number of ROS. Since cells exposed to this agent showed approximately two and a half times more ROS than cells not exposed to peroxide (NC).

Figure 3C demonstrates that, following peroxide exposure, the activities of caspases 3 and 9 (key apoptotic markers) increased significantly compared to the negative control group. These results suggest that 3T3-L1 cells undergo apoptosis under the experimental conditions, thereby explaining the observed decrease in MTT reduction.

Previous studies corroborate these findings. Teramoto et al. [44] reported that, in human fibroblast HFL-1 cells, lower concentrations of H_2_O_2_ (10–100 µM) preferentially induce apoptosis, whereas higher concentrations (1.0–10 mM) promote necrosis. Although 3T3-L1 cells are derived from murine fibroblasts, the results obtained in the present study are consistent with those of Teramoto et al., reinforcing that at the concentration employed (15 µM), H_2_O_2_ predominantly induces apoptosis in this cellular model.

The compound quercetin (10 µM), a well-recognized reference antioxidant, was employed as a positive control for the assessment of cellular protection. The administration of quercetin effectively mitigated oxidative damage induced by peroxide, as demonstrated by the absence of statistically significant differences in MTT reduction capacity between the negative control and the quercetin-treated cells (Figure 3A), as well as in ROS levels (Figure 3B) and caspase activity (Figure 3C).

The polysaccharide F1.5 similarly conferred protection against oxidative stress at the two highest concentrations examined (0.5 and 1.0 mg/mL). Notably, at the 1.0 mg/mL concentration, the MTT reduction values (Figure 3A), the ROS levels (Figure 3B), and the caspase activity (Figure 3C) were statistically comparable to those observed in cells not exposed to peroxide (NC).

Determining the appropriate dosage is a critical consideration when supplementing with natural antioxidants, as these exogenous compounds can exert pro-oxidant effects depending on concentration [45]. Such dual behavior has been reported for certain sulfated polysaccharides derived from algae, including some fucoidans, which induce ROS-mediated apoptosis in MCF-7 cells at concentrations of 1.0 mg/mL [46,47] and 820 µg/mL [47], via both caspase-dependent and caspase-independent mechanisms. The findings obtained with F1.5 not only demonstrate its protective effect against peroxide-induced oxidative stress but also indicate that, at higher concentrations, it does not exhibit pro-oxidant activity within the cellular environment.

### 2.6. Lipid Peroxidation Analysis

**Figure 4:**
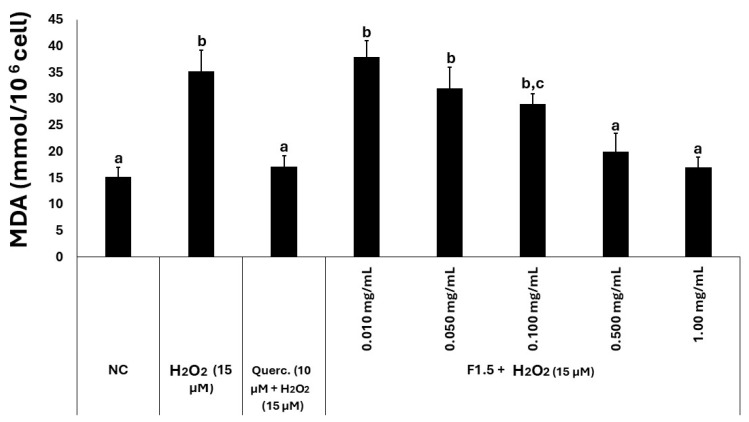
F1.5 reduces lipid peroxidation marker MDA in H_2_O_2_-challenged 3T3-L1 cells. Levels of malondialdehyde (MDA) were determined in 3T3-L1 cells exposed to H_2_O_2_ and F1.5 or quercetin (Querc.) as a positive control. The values are presented as the mean ± standard deviation (SD) from three independent experiments. NC stands for negative control (cells treated with DMEM + FBS only). Different lowercase letters (a, b, c) indicate significant differences among the samples (*p* < 0.05).

Malondialdehyde (MDA) levels were quantified to assess the extent of free radical generation in 3T3-L1 cells (Figure 4). The concentration of MDA measured in the control group was approximately 15 mmol/10^6^ cells, which was considered the physiological baseline. In the presence of hydrogen peroxide, MDA levels increased approximately twofold, indicating that the compound induced oxidative damage. Treatment with quercetin (10 µM) resulted in MDA concentrations that were statistically comparable to those of the control group. Similarly, when cells were exposed to both peroxide and F1.5, MDA levels progressively decreased with increasing concentrations of F1.5, ultimately reaching values that were not statistically different from those observed in the control group.

### 2.7. Assessment of the Cell Antioxidant Status

**Figure 5:**
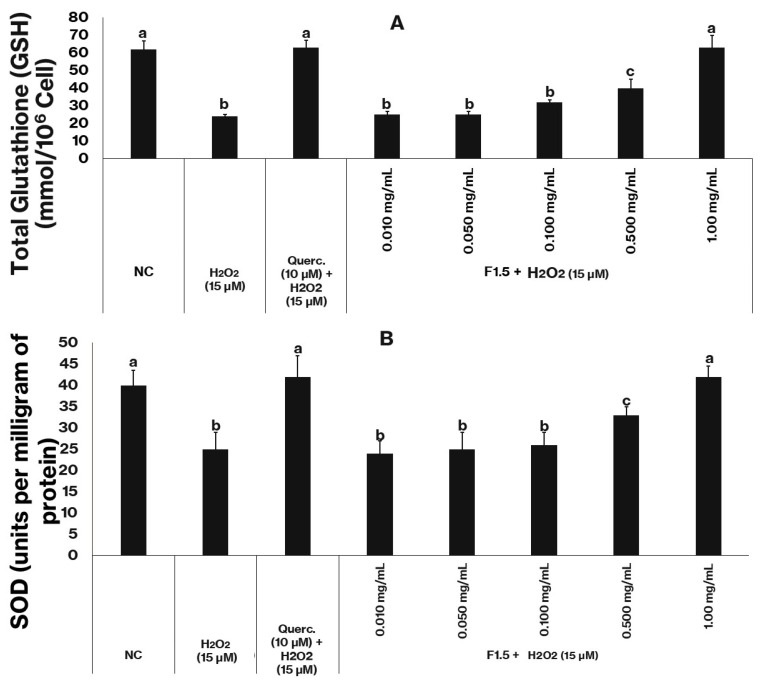
F1.5 enhances the antioxidant defense system in oxidatively stressed 3T3-L1 cells. (**A**)—F1.5 increase GSH levels in 3T3-L1 Cells. (**B**)—F1.5 elevates total superoxide dismutase (SOD) activity in 3T3-L1 Cells. Querc = quercetin used as the positive control. NC stands for negative control (cells treated with DMEM + FBS only). The values are presented as the mean ± standard deviation (SD) from three independent experiments. Different lowercase letters (a, b, c) indicate significant differences among the samples (*p* < 0.05).

The intracellular antioxidant defense status of 3T3-L1 cells subjected to oxidative stress was evaluated through the quantification of reduced glutathione (GSH) and superoxide dismutase (SOD) activity (Figure 5).

Cells from the negative control group exhibited basal GSH levels of approximately 60 (mmol/10^6^ cells) (Figure 5A) and SOD activity around 40 U/mg of protein (Figure 5B), values considered representative of normal redox homeostasis. Exposure to hydrogen peroxide (15 µM) markedly reduced both parameters: GSH content decreased to about one third of the control level, and SOD activity fell by nearly 40%. These declines confirm that peroxide exposure promotes intense oxidative stress, resulting in consumption of non-enzymatic antioxidants and inactivation of antioxidant enzymes.

In contrast, treatment with quercetin (10 µM), used as a positive control, effectively prevented oxidative damage, maintaining both GSH and SOD values statistically similar to those of the control group. When cells were treated with F1.5 in the presence of H_2_O_2_, a concentration-dependent protective pattern emerged. At lower concentrations (0.01–0.10 mg/mL), GSH and SOD levels remained below control values, indicating partial protection. However, at 0.5 mg/mL, both parameters increased substantially, and at 1.0 mg/mL, they reached values comparable to those of untreated cells, demonstrating full recovery of redox balance.

These results clearly indicate that F1.5 attenuates the oxidative damage induced by H_2_O_2_ by preserving both enzymatic and non-enzymatic antioxidant systems. The dose-dependent recovery of GSH levels suggests that F1.5 prevents excessive glutathione oxidation and maintains thiol homeostasis, either by directly scavenging reactive oxygen species or by sparing endogenous GSH. In parallel, the restoration of SOD activity implies that F1.5 prevents enzyme inactivation or depletion, possibly by limiting superoxide accumulation or by protecting metal cofactors essential for SOD function.

Taken together with the previously in vitro assays and the observed reduction in MDA levels and the recovery of mitochondrial viability (MTT assay), these findings indicate that F1.5 exerts a broad cytoprotective effect under oxidative stress conditions. Its ability to restore both GSH and SOD aligns with the profile of compounds that act not only as direct free radical scavengers but also as stabilizers of the cellular redox network. The similar efficacy between F1.5 (1.0 mg/mL) and quercetin (10 µM) underscores its potential as a bioactive polysaccharide with antioxidant capacity. The protective mechanisms of F1.5 may involve radical neutralization, metal ion chelation, and preservation of redox-sensitive enzymes, thereby preventing oxidative cascades that culminate in lipid peroxidation and cell death.

### 2.8. Antiadipogenic Effect of F1.5

**Figure 6:**
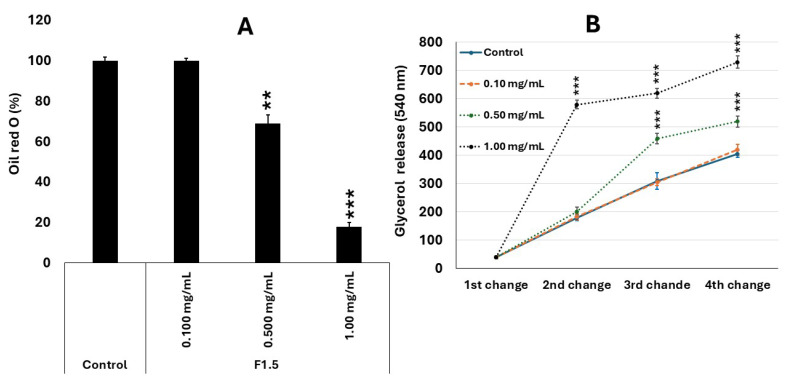
F1.5 modulates lipid metabolism in 3T3-L1 cells by inhibiting lipid accumulation and promoting lipolysis. (**A**)—F1.5 Inhibits intracellular lipid accumulation in 3T3-L1 adipocytes. (**B**)—F1.5 Promotes Lipolysis by Increasing Glycerol Release from 3T3-L1 Adipocytes Over Time. The values are presented as the mean ± standard deviation (SD) from three independent experiments. Control = cells treated with DMEM + FBS only. ** *p* < 0.01 and *** *p* < 0.001 indicate significant differences compared with the control.

Treatment of 3T3-L1 cells with the polysaccharide F1.5 promoted a significant inhibition of adipogenic differentiation, as evidenced by Oil Red O staining (Figure 6A) and the release of glycerol into the culture medium (Figure 6B). Cells in the control group displayed intense lipid staining, consistent with the formation of mature adipocytes, whereas those treated with 0.1 mg/mL of F1.5 exhibited a similar profile, with no significant changes in lipid accumulation. In contrast, at concentrations of 0.5 and 1.0 mg/mL, a progressive and dose-dependent reduction in intracellular lipid content was observed, reaching approximately 70% and 20% of the control values, respectively.

This pattern was accompanied by a corresponding decrease in extracellular glycerol levels, an indirect marker of lipolysis. While the control group showed a gradual increase in glycerol concentration throughout the medium changes, treatment with F1.5 significantly reduced these values, particularly at doses of 0.5 and 1.0 mg/mL. The lower release of glycerol suggests that F1.5 impaired triglyceride accumulation and/or metabolism, thereby reducing the formation of metabolically active adipocytes.

These results indicate that F1.5 exerts an inhibitory effect on adipogenesis in 3T3-L1 cells, interfering with both the differentiation phase and the lipid metabolism of mature adipocytes. Mechanistically, bioactive polysaccharides, especially sulfated ones of marine origin, have been described as negative modulators of adipogenesis, acting through the regulation of transcription factors such as PPARγ, C/EBPα, and SREBP-1c, which are essential for lipid accumulation during differentiation [48,49]. Inhibition of these pathways leads to a reduction in the expression of lipogenic enzymes, such as acetyl-CoA carboxylase (ACC) and fatty acid synthase (FAS), resulting in lower triglyceride deposition [50]. However, it is important to note that the antiadipogenic mechanism of F1.5 has not yet been elucidated, and the involvement of these pathways remains a hypothesis to be experimentally addressed in future studies.

Furthermore, the antioxidant effect previously demonstrated for F1.5 may contribute to the observed phenomenon. ROS are recognized as important mediators of adipogenesis, acting in the activation of signaling pathways that promote preadipocyte differentiation [51,52]. Thus, the ability of F1.5 to reduce intracellular oxidative stress may limit the redox signaling required for the activation of these pathways, leading to decreased lipid accumulation and glycerol production.

Indeed, the results obtained from antioxidant assays support this hypothesis: F1.5 significantly increased SOD and GSH levels and reduced MDA concentrations, restoring redox homeostasis in cells exposed to hydrogen peroxide. This protective effect against oxidative stress likely acts synergistically with the modulation of lipogenic pathways, explaining the reduced adipocyte differentiation observed. Collectively, these findings suggest that F1.5 exerts a dual, interconnected action, antioxidant and antiadipogenic, which may confer promising potential for the prevention of metabolic disorders associated with lipid accumulation and oxidative imbalance.

## 3. Materials and Methods

### 3.1. Materials

Nitro blue tetrazolium (NBT), toluidine blue, 1,3-diaminopropane, acetone, Folin–Ciocalteau, Coomassie brilliant blue R-250, 2,2′,2″,2‴-(Ethane-1,2-diyldinitrilo) tetra-acetic acid (EDTA), ascorbic acid, methionine, ammonium molybdate, quercetin, Dulbecco’s Modified Eagle Medium (DMEM), and Roswell Park Memorial Institute (RPMI) medium were all purchased from Sigma (St. Louis, MO, USA). Methanol, ethanol, acetone, acetic acid, sulfuric acid, and N-cetyl-N,N,N-trimethylammonium bromide (CTV) were purchased from CRQ (São Paulo, SP, Brazil). Sterile fetal bovine serum (FBS) was purchased from Cultilab (Campinas, SP, Brazil). Penicillin and streptomycin were obtained from Thermo Fisher Scientific (Waltham, MA, USA). All other solvents and chemical products used in this study were of analytical-grade purity.

Specimens of *Dictyopteris delicatula* J.V. Lamouroux, *Dictyota menstrualis* (Hoyt) Schnetter, *Dictyota mertensii* (Martius) Kützing, *Spatoglossum schröederi* (C. Agardh), *Caulerpa cupressoides* (Vahl) C. Agardh, *Caulerpa prolifera* (Forsskål) J.V. Lamouroux, *Caulerpa sertularioides* (S.G. Gmelin) M.A. Howe, *Caulerpa racemosa* (Forsskål) J. Agardh, *Codium isthmocladum* Vickers, *Udotea flabellum* (J.Ellis & Solander), and *Gracilaria caudata* J. Agardh were collected along the coastal region of Nísia Floresta (05°58′23″ S, 35°04′97″ W), Rio Grande do Norte, Brazil. In addition, specimens of *Gracilaria birdiae* E.M. Plastino & E.C. Oliveira, *Dictyopteris justii* J.V. Lamouroux, and *Sargassum filipendula* C. Agardh were collected in Rio do Fogo, Rio Grande do Norte (05°16′22″ S, 35°22′58″ W), Brazil. Species identification was performed based on morphological characteristics according to established taxonomic criteria [53]. The collection of *G. birdiae* was conducted under authorization from the Brazilian National System for the Management of Genetic Heritage and Associated Traditional Knowledge (SISGEN, registration no. A72AD2B), while the collection of the other algal species was carried out under SISGEN authorization no. A0D4240.2.1.1.

PANC-1 (epithelioid carcinoma cells from human pancreatic duct) cells, Hela (human cervical adenocarcinoma cells), CHO (Chinese hamster ovary cells), RAW 264.7 (murine macrophage cells), 3T3-L1 (clonal line of preadipocytes derived from mouse embryonic fi-broblasts), and PC3 (human prostate cancer cells) were maintained in DMEM or RPMI medium (for PC3 cells) supplemented with fetal bovine serum, streptomycin, and penicillin (all cells were purchased from the Rio de Janeiro Cell Bank (BCRJ), located at the Federal University of Rio de Janeiro (UFRJ), Brazil). The culture medium was refreshed every three days. For subculturing, adherent cells were detached using a trypsin–EDTA solution, except for RAW cells, which were harvested by gentle scraping.

### 3.2. Extraction of Sulfated Polysaccharide-Rich Extracts (SPREs)

The extraction of sulfated polysaccharide-rich extracts (SPREs) was carried out following the procedure adapted from Rocha et al. [54]. Freshly collected seaweeds were immediately dried in a ventilated oven at 50 °C and then finely milled. The resulting powder was defatted with ethanol to remove pigments and lipophilic compounds. Subsequently, 100 g of the dried and defatted material was suspended in 500 mL of 0.25 M NaCl, and the pH was adjusted to 8.0 with NaOH. Enzymatic hydrolysis was performed by adding an alkaline protease preparation (Prolav 750; Prozyn Biosolutions, São Paulo, Brazil) at a concentration of 15 mg/g of dry seaweed, followed by incubation at 60 °C for 18 h. The suspension was then centrifuged (8000× *g*, 30 min, 4 °C), and the resulting pellet, corresponding to the SPRE, was collected, dried under reduced pressure, weighed, and stored in a dry, dark environment. For subsequent experiments, the SPREs were dissolved in distilled water.

### 3.3. Determination of Sulfate, Protein, Monosaccharide, and Phenolic Contents

The sulfate concentration in the samples was determined following the gelatin–barium method of Dodgson and Price [55], by measuring absorbance at 500 nm with sodium sulfate used for calibration. Protein levels were assessed according to the Bradford assay [56], with readings taken at 595 nm and bovine serum albumin serving as the standard. The total phenolic content was evaluated using the colorimetric Folin–Ciocalteu procedure as outlined by Singleton and Rossi [57]; absorbance was recorded at 765 nm, and gallic acid was used as the reference compound.

To determine the best polysaccharide acid hydrolysis conditions when using HCl, that is, where polymer degradation occurs without destroying the monosaccharides released by this degradation, polysaccharides from F1.5, F2.0, and F3.0 were hydrolyzed with HCl at concentrations of 0.5 M, 1 M, 2 M, and 4 M, for 30 min, 1 h, 2 h, and 4 h, respectively. A temperature of 100 °C was maintained for all hydrolysis conditions. The material was later neutralized, dried, and resuspended in water, and reducing sugars were determined using the Somogyi–Nelson method [58]. In all cases, the best hydrolysis condition was 2 M HCl for 2 h at 100 °C. Thus, polymers were hydrolyzed using those conditions and their sugar composition was determined using a LaChrom Elite^®^ HPLC system (Hitachi Co., Tokyo, Japan) with a refractive index detector (RI detector model L-2490). A LichroCART^®^ 250-4 column (250 mm × 40 mm) packed with Lichrospher^®^ 100 NH2 (5 µm) was coupled to the system (Both columns are from Merck Co., Darmstadt, Germany). The sample mass used was 0.2 mg and the analysis time was 25 min. The following sugars were analyzed as a reference: arabinose, fructose, fucose, galactose, glucose, glucosamine, glucuronic acid, mannose, mannuronic acid, rhamnose, and xylose.

### 3.4. Extraction of Sulfated Polysaccharide from G. caudata SPRE

For the separation of polysaccharides present in the SPRE from *G. caudata*, the extract was dissolved in 0.25 M NaCl (1 g in 100 mL) under continuous stirring at room temperature for 12 h. The insoluble material was then removed by centrifugation (8000× *g*, 30 min, 4 °C). The pellet was discarded, and 150 mL of cold acetone was added to the supernatant until turbidity appeared, indicating polysaccharide precipitation. The mixture was kept at 4 °C for 12 h, protected from light, to allow complete precipitation. After centrifugation (8000× *g*, 30 min, 4 °C), the precipitate, designated as F1.5, was dried under reduced pressure, weighed, and stored for further analyses. Additional acetone (50 mL) was added to the supernatant until a new precipitate formed. The same procedure was repeated, and the resulting pellet was designated as F2.0. A further addition of 100 mL of cold acetone to the remaining supernatant produced another precipitate, which was processed as described above and designated as F3.0. The subsequent addition of up to 200 mL of acetone did not produce any new precipitate; therefore, the remaining solution was discarded and sent for incineration. For subsequent assays, F1.5, F2.0, and F3.0 were dissolved in distilled water at a concentration of 10 mg/mL, and these solutions were used as stock samples.

### 3.5. Molecular Weight Determination

The molecular weight of the F1.5, F2.0, and F3.0 was assessed using high-performance size-exclusion chromatography (HPSEC) with a TSK-Gel^®^ 3000 column (30 cm × 0.75 cm) from Sigma-Aldrich (St. Louis, MO, USA), operated on a system from GE Healthcare Biosciences (Pittsburgh, PA, USA). The mobile phase consisted of 0.2 M sodium chloride in 0.05 M acetate buffer. Chromatographic runs were carried out at 60 °C with a flow rate of 1.0 mL/min. The column was calibrated using dextran standards with molecular weights of 10, 47, 74, and 147 kDa, also obtained from Sigma-Aldrich (St. Louis, MO, USA). A refractive index detector was used to monitor the eluted samples.

### 3.6. Agarose Gel Electrophoresis Analysis

Agarose gel electrophoresis was performed according to the method described by Dietrich and Dietrich [59] to verify the presence or absence of sulfated polysaccharides (SPs) in the samples. Gels were prepared at a concentration of 0.6% (*w*/*v*) in 0.05 M 1,3-diaminopropane–acetate (PDA) buffer and cast onto glass slides, where wells were formed for sample loading. Each well received 1 μg of the sample. Electrophoretic separation was conducted at 4 °C in a refrigerated chamber under a constant current of 0.3 A and an applied voltage of 110 V, allowing migration from the cathode to the anode.

Following electrophoresis, gels were immersed in 0.1% (*w*/*v*) cetyltrimethylammonium bromide (CTV) for approximately 2 h to facilitate SP precipitation. The gels were then air-dried and stained with 0.1% (*w*/*v*) toluidine blue. Excess dye was removed using a decolorizing solution composed of 1% (*v*/*v*) acetic acid, 50% (*v*/*v*) ethanol, and 49% (*v*/*v*) distilled water. Finally, the gels were dried again at room temperature before visualization and analysis.

### 3.7. In Vitro Antioxidant Assays

The antioxidant tests were carried out to compare the antioxidant capacity of the sulfated polysaccharide from *G. caudata*. Tests for total antioxidant capacity, reducing power, superoxide and hydroxyl radical scavenging, and iron chelating were carried out as described by Silva et al. [60].

#### 3.7.1. Hydroxyl Radical Scavenging Activity Assay

The hydroxyl radical scavenging activity was evaluated using a reaction system composed of 10 mM FeSO_4_·7H_2_O, 10 mM EDTA, 2 mM sodium salicylate, and 30% H_2_O_2_. A 150 mM phosphate buffer (pH 7.4) was used as the negative control, and gallic acid served as the positive control. Test samples were prepared at a concentration of 10 mM and incubated with the reaction mixture for 1 h at 37 °C. After incubation, the absorbance was measured at 510 nm using a microplate reader to determine the hydroxyl radical scavenging capacity. The scavenging activity (%) was calculated as follows:

$$\%\mathrm{Scavenging}=[\frac{\left(Ac-A\right)}{\left(Ac-Ab\right)}]\times 100$$

where *Ac* represents the absorbance of the control, *Ab* that of the blank, and *A* the absorbance of the sample.

#### 3.7.2. Superoxide Anion Scavenging Assay

The superoxide radical scavenging activity was determined based on the inhibition of nitroblue tetrazolium (NBT) photoreduction in the riboflavin–light–NBT system. Under illumination, riboflavin reduces oxygen to generate superoxide anions, which subsequently reduce NBT to form a colored product. Compounds with superoxide scavenging capacity inhibit this reduction reaction.

For the assay, 200 µL of methionine (65 mM), 200 µL of EDTA (0.5 mM), 200 µL of NBT (0.375 mM), and 200 µL of riboflavin (0.5 mM) were added to each sample. The blank consisted of buffer only and was protected from light during riboflavin addition. All reaction tubes, except for the blank, were placed in a Styrofoam box lined with aluminum foil and exposed to fluorescent light for 15 min. After incubation, absorbance was measured at 560 nm using a microplate reader. The scavenging activity (%) was calculated as follows:

$$\%\mathrm{Scavenging}=[\frac{\left(Ac-A\right)}{\left(Ac-Ab\right)}]\times 100$$

where *Ac* represents the absorbance of the control, *Ab* that of the blank, and *A* the absorbance of the sample.

#### 3.7.3. Ferrous Ion (Fe^2+^) Chelation Assay

The ferrous ion chelating ability was evaluated using a colorimetric method based on the formation of a colored complex between Fe^2+^ and ferrozine. The assay was performed in 96-well microplates, where FeCl_2_ (2 mM) and ferrozine (5 mM) were added to the samples. The mixture was gently shaken and incubated for 10 min at 37 °C. Absorbance was recorded at 562 nm using a spectrophotometer. Chelating activity (%) was calculated according to the following equation:

$$\%\mathrm{Chelation}=[\frac{\left(Ab-Aa\right)}{Ab}]\times 100$$

where *Ab* corresponds to the absorbance of the blank and *Aa* to the absorbance of the sample.

#### 3.7.4. Total Antioxidant Capacity (TAC) Assay

The total antioxidant capacity was determined by incubating the samples with a reagent solution containing 0.6 M sulfuric acid, 28 mM sodium phosphate, and 4 mM ammonium molybdate. The mixture was incubated in a water bath at 100 °C for 90 min. After cooling to room temperature, the absorbance was measured at 695 nm using a spectrophotometer. Ascorbic acid was used as the reference standard, and the results were expressed as milligrams of ascorbic acid equivalents (mg AAE) per gram of sample.

#### 3.7.5. Reducing Power Assay

The reducing power of the samples was assessed by incubating them with 0.2 M phosphate buffer (pH 6.6) and 1% (*w*/*v*) potassium ferricyanide at 50 °C for 20 min. The reaction was stopped by adding 10% (*w*/*v*) trichloroacetic acid (TCA), followed by the addition of 0.1% (*w*/*v*) ferric chloride. Ascorbic acid was used as a standard. Absorbance was measured at 700 nm using a spectrophotometer, and the results were expressed as the percentage of reducing power relative to ascorbic acid, according to the equation:

$$\%\mathrm{Reducing}\;\mathrm{Power}=[\frac{\left(Ac-A\right)}{\left(Ac-Ab\right)}]\times 100$$

where *Ac* denotes the absorbance of the control, *Ab* that of the blank, and *A* the absorbance of the sample.

### 3.8. Assessment of Cellular Cytotoxicity

Cells were seeded in 96-well plates at a density of 5 × 10^3^ cells per well and cultured for 24 h under standard conditions (37 °C, 5% CO_2_) in DMEM or RPMI medium (in case of PC3 cells) supplemented with 10% FBS and antibiotics (penicillin and streptomycin). After 24 h, the culture medium was replaced with serum-free medium to induce cell starvation under the same conditions. The cells were then treated with media containing varying concentrations of samples. Following the treatment period, the medium was removed, and MTT (3-[4,5-dimethylthiazol-2-yl]-2,5-diphenyltetrazolium bromide) solution (1 mg/mL) was added to each well and incubated for 4 h. After this period, the MTT solution was removed and replaced with DMSO to dissolve the formazan crystals. Absorbance was measured at 570 nm. The results were expressed as a percentage of MTT reduction, with the untreated control cells set as 100%.

### 3.9. Assessment of Oxidative Stress Induced by Hydrogen Peroxide (H_2_O_2_)

This assay evaluates the capacity of the test samples to shield cells from damage induced by H_2_O_2_ under two different conditions: preventive and concomitant. The methodology for this assay was adapted from the procedures described by Fernandes-Negreiros et al. [61].

The concentrations of F1.5 evaluated in this assay (0.01–1 mg/mL) were selected based on the absence of cytotoxicity observed in the initial cell viability tests, which confirmed that F1.5 is non-toxic up to 1 mg/mL (Figure 2). This range is also consistent with concentrations commonly used for sulfated polysaccharides demonstrating antioxidant and metabolic activity in cellular models [49,62,63]. Choosing this range additionally prevents exposure to excessively high concentrations that may induce pro-oxidant responses previously reported for antioxidant agents [64] including sulfated polysaccharides [65].

Following the protocols outlined in Section 3.7, the cells were cultured, allowed to adhere, and then subjected to starvation. After this, they were treated with 15 µM H_2_O_2_ along with samples for 2 h. Then, the medium containing H_2_O_2_ and the samples was removed, and the cells were then incubated under standard culture conditions for another 24 h. Subsequently, toxicity was evaluated using the MTT reduction assay, as detailed in Section 3.7. The positive control group was treated solely with 15 µM H_2_O_2_, while the negative control was treated with culture medium containing FBS. The results were expressed as a percentage reduction in MTT, with the positive control set as 100%.

### 3.10. Intracellular ROS Detection Assay

3T3-L1 cells were seeded into 96-well plates with transparent and dark bottoms at a density of 2 × 10^4^ cells per well and cultured for 24 h under standard conditions (37 °C, 5% CO_2_) in DMEM supplemented with 10% fetal bovine serum (FBS) and antibiotics (penicillin and streptomycin). After 24 h, the culture medium was replaced with serum-free DMEM to induce cell starvation under the same incubation conditions. The cells were then washed twice with phosphate-buffered saline (PBS) pre-warmed to 37 °C. Subsequently, hydrogen peroxide (H_2_O_2_, 15 µM) and the test samples at different concentrations were added simultaneously. The negative control consisted of cells exposed only to the culture medium. After two hours of incubation, the medium was removed, and the cells were washed twice with pre-warmed PBS (37 °C). Then, 100 µL of serum-free DMEM containing 10 µM 2′,7′-dichlorodihydrofluorescein diacetate (H_2_DCF-DA) was added to each well, and the plate was incubated in the dark at 37 °C for 45 min. Fluorescence intensity was subsequently measured using a microplate reader (GloMax^®^ Multi Detection System, Promega, WI, USA) with excitation at 490 nm and emission between 510 and 570 nm. The fluorescence intensity of the negative group (NC) was considered as 100%, and the fluorescence of the treated groups was expressed as a percentage relative to the control.

### 3.11. Caspase-3 and -9 Activity Assays

The activity of caspase-3 and caspase-9 was evaluated to assess apoptotic induction in 3T3-L1 cells. Briefly, 3T3-L1 cells (1 × 10^6^ cells/mL) were seeded into 6-well plates containing 1 mL of DMEM supplemented with 10% fetal bovine serum (FBS) and antibiotics. After 24 h of incubation under standard culture conditions (37 °C, 5% CO_2_), the cells were washed with pre-warmed phosphate-buffered saline (PBS, 37 °C). Subsequently, 1 mL of DMEM supplemented with 10% FBS containing H_2_O_2_, quercetin, and/or F1.5 was added. After 120 min, the medium was replaced with fresh DMEM and the cells were further incubated for 24 h.

Following treatment, the cells were washed with ice-cold PBS and scraped into 200 µL of lysis buffer [50 mM Tris-HCl (pH 7.4), 1% Tween 20, 0.25% sodium deoxycholate, 150 mM NaCl, 1 mM EDTA, 1 mM Na_3_VO_4_, and 1 mM NaF], supplemented with protease inhibitors [1 µg/mL aprotinin, 10 µg/mL leupeptin, and 1 mM 4-(2-aminoethyl)benzenesulfonyl fluoride]. Cell lysates were kept on ice for 2 h. Untreated cells were processed under identical conditions and served as the negative control (NC). After centrifugation, protein concentrations in the supernatants were quantified using the Bradford method [56], with bovine serum albumin as the standard.

Caspase-3 and caspase-9 proteolytic activities were determined using a commercial Caspase Activation Kit (Invitrogen, São Paulo, Brazil) according to the manufacturer’s protocol. In brief, 50 µL of cell lysate was combined with 50 µL of 2× reaction buffer containing 10 µL of 1 M dithiothreitol and 5 µL of 4 mM synthetic tetrapeptide substrates—Asp-Glu-Val-Asp-pNA for caspase-3 and Leu-Glu-His-Asp-pNA for caspase-9—in a 96-well plate. The mixtures were incubated for 2 h at 37 °C in the dark. Active caspases cleave the substrates, releasing the chromophore p-nitroanilide (pNA), which was detected spectrophotometrically at 405 nm. Apoptotic cell lysates containing active caspases exhibited significantly higher absorbance values compared with non-apoptotic controls.

### 3.12. Superoxide Dismutase (SOD) Activity Assay

Superoxide dismutase (SOD) activity was quantified using a commercial SOD Activity Kit (Enzo Life Sciences, Farmingdale, NY, USA). The method is based on the capacity of SOD to neutralize superoxide anions generated by the xanthine/xanthine oxidase system, thereby inhibiting the reduction in the water-soluble tetrazolium salt WST-1 to its formazan derivative.

Briefly, 3T3-L1 cells (1 × 10^6^ cells/mL) were seeded into 6-well plates containing 1 mL of DMEM supplemented with 10% FBS and antibiotics. After 24 h of incubation under standard culture conditions (37 °C, 5% CO_2_), the cells were washed with pre-warmed phosphate-buffered saline (PBS, 37 °C). Subsequently, 1 mL of DMEM supplemented with 10% FBS containing H_2_O_2_, quercetin, and/or F1.5 was added. After 2 h of treatment, the cells were washed with ice-cold 1× PBS and lysed according to the manufacturer’s instructions. The resulting supernatants were collected, and total SOD activity was determined spectrophotometrically at 450 nm. The enzyme activity was expressed as units per milligram of protein, calculated from a standard SOD calibration curve.

### 3.13. Glutathione (GSH) Assay

Total intracellular glutathione levels were quantified in 3T3-L1 cells (5 × 10^6^ cells/well) 2 h after oxidative stress induction, as described in Section 3.11. The cells were washed twice with ice-cold 1× PBS, collected, resuspended in PBS, and centrifuged at 3000× *g* for 5 min at 4 °C. The resulting pellet was diluted in 50% trichloroacetic acid (Vetec, São Paulo, Brazil) and centrifuged for 15 min at 3000× *g* and 4 °C. The obtained supernatant was then mixed with an equal volume of 0.4 M Tris buffer containing 0.01 M 5,5′-dithiobis(2-nitrobenzoic acid) (DTNB; Sigma-Aldrich Co., St. Louis, MO, USA). The absorbance was measured at 412 nm, and total GSH levels were expressed as nmol per 10^6^ cells.

### 3.14. Malondialdehyde (MDA) Assay

Lipid peroxidation was evaluated by measuring malondialdehyde (MDA) formation through the thiobarbituric acid reactive substances (TBARS) method. Briefly, cells treated under the same conditions described above were homogenized in 20 mM Tris-HCl buffer and centrifuged at 3000× *g* for 15 min at 4 °C. To each 150 µL of supernatant, the chromogenic reagent, 10.3 mM 1-methyl-2-phenylindole in acetonitrile (3:1, *v*/*v*), and concentrated HCl (37%) were added. The samples were incubated at 45 °C for 40 min and subsequently centrifuged at 3000× *g* for 15 min at 4 °C. Absorbance was recorded at 586 nm using a spectrophotometer, and MDA levels were expressed as nmol per 10^6^ cells.

### 3.15. Differentiation of 3T3-L1 Cells

Mouse preadipocyte 3T3-L1 cells, upon reaching confluence, were induced to differentiate using an adipogenic differentiation medium (ADM) consisting of DMEM supplemented with 1 μM dexamethasone, 0.5 mM isobutylmethylxanthine (IBMX), 10 μg/mL insulin, and different concentrations of F1.5. On the third day, the induction medium was replaced with an adipocyte maintenance medium composed of DMEM containing 4.5 g/L glucose, 10% fetal bovine serum (FBS), and 10 μg/mL insulin. The maintenance medium was renewed every three days until day 15.

The ability of F1.5 to inhibit adipogenic differentiation (antiadipogenic effect) was evaluated by comparing the differentiation of 3T3-L1 cells induced in the absence (control) or presence of F1.5, which was added concurrently with the differentiation medium. All culture media contained 100 U/mL penicillin and 100 μg/mL streptomycin.

### 3.16. Antiadipogenic Activity

#### 3.16.1. Quantification of Cellular Differentiation Using Oil Red O Staining

Fifteen days after differentiation induction, intracellular lipid accumulation was evaluated by Oil Red O staining. Briefly, cells were washed twice with PBS and fixed with 3.7% formaldehyde for 10 min. Fixed cells were then stained with 0.2% Oil Red O in isopropanol for 1 h. Excess dye was removed by sequential washing with 70% ethanol and distilled water. The stained lipid droplets were dissolved in isopropanol, and absorbance was measured spectrophotometrically at 510 nm. The optical density of cells treated with the differentiation medium alone (MDI) was considered as 100% relative lipid content. Results were expressed as relative lipid accumulation for each experimental group.

#### 3.16.2. Measurement of Free Glycerol

Free glycerol content was quantified using a commercial Free Glycerol Assay Kit (Sigma-Aldrich Co., St. Louis, MO, USA), according to the manufacturer’s instructions. The free glycerol reagent was prepared at room temperature prior to use. The reaction mixtures were prepared as follows: Negative control (NC): 10 μL distilled water + 400 μL reagent; Standard: 5 μL glycerol + 400 μL reagent; Samples: 10 μL of F1.5 solution + 400 μL reagent.

The mixtures were gently inverted to ensure homogeneity and incubated in a water bath at 37 °C for 5 min. Absorbance was measured at 540 nm to determine the enzymatic hydrolysis of triglycerides by lipase, generating glycerol and free fatty acids. The increase in absorbance at 540 nm was directly proportional to the concentration of free glycerol.

### 3.17. Statistical Analysis

Data were expressed as mean ± standard deviation from at least three independent experiments, each performed in triplicate. Statistical analysis was conducted using one-way ANOVA, followed by Bonferroni’s post hoc test, with a significance level set at *p* < 0.05. All statistical tests were performed using GraphPad Prism 5 software (GraphPad Software, San Diego, CA, USA).

## 4. Conclusions

In conclusion, the sulfated polysaccharide F1.5 isolated from *Gracilaria caudata* exhibited potent antioxidant activity and effectively protected 3T3-L1 cells against H_2_O_2_-induced oxidative stress, in addition to inhibiting adipogenic differentiation in vitro. These results highlight F1.5 as a promising natural compound with potential application in preventing oxidative and metabolic disorders associated with lipid accumulation. However, the present study was limited to in vitro assays, and therefore, the mechanisms of action and physiological relevance remain to be fully elucidated.

Future investigations will include the evaluation of key adipogenic regulators, such as PPARγ, C/EBPα, SREBP-1c, ACC, and FAS, using Western blot and RT-qPCR, alongside the use of rosiglitazone or another drug as a positive pharmacological control, to better clarify the antiadipogenic mechanism of F1.5. Additionally, advanced structural characterization of F1.5 using techniques such as FTIR, NMR spectroscopy, methylation analysis, and mass spectrometry will be carried out to reveal its structural motifs and their relationship to bioactivity. Subsequent studies should also involve in vivo validation using appropriate animal models, as well as assessments of bioavailability, safety, and formulation stability.

Finally, based on the reviewer’s valuable suggestion, future research will also explore whether the stronger ROS-scavenging profile of F2.0 translates into distinct biological responses at lower doses. Comparative cellular assays between F1.5 and F2.0 will evaluate dose-dependent effects on ROS homeostasis, apoptosis, and metabolic signaling in normal versus tumor cells, to determine whether different antioxidant strengths correlate with selective cytotoxicity or therapeutic potential.

Together, these future efforts will be essential to confirm the therapeutic potential of *G. caudata* polysaccharides and to support their development as candidates for nutraceutical or biomedical applications.

## Figures and Tables

**Table 1:** Chemical composition and antioxidant activity of sulfated polysaccharide-rich extracts from different seaweeds.

	Seaweed	Sulfate/Sugar (%/%)	Protein (%)	Phenolic Compounds (%)	TAC *
Brown seaweed	*Dictyopteris justii*	0.68	0.3 ± 0.1	0.8 ± 0.2	31.5 ± 3.1 ^a^
	*Dictyopteris delicatula*	0.41	0.3 ± 0.1	1.9 ± 0.2	27.8 ± 0.8 ^a^
	*Dictyota menstruallis*	0.20	0.4 ± 0.2	0.7 ± 0.1	27.3 ± 3.1 ^a^
	*Dictyota mertensis*	0.40	0.1 ± 0.1	0.3 ± 0.1	19.2 ± 1.6 ^b^
	*Sargassum filipendula*	0.45	0.2 ± 0.1	0.7 ± 0.2	33.4 ± 1.7 ^a^
	*Spatoglossum schröederi*	0.24	0.3 ± 0.2	0.8 ± 0.1	17.2 ± 1.5 ^b^
	*Dictyota mertensis*	0.45	0.2 ± 0.1	0.5 ± 0.2	20.2 ± 1.5 ^b^
Green seaweed	*Caulerpa cupresoides*	0.29	0.2 ± 0.1	0.8 ± 0.2	20.4 ± 1.1 ^b^
	*Caulerpa prolifera*	1.19	0.1 ± 0.1	0.7 ± 0.5	21.7 ± 2.1 ^b^
	*Caulerpa sertularioides*	0.80	0.1± 0.1	0.9 ± 0.2	20.9 ± 1.6 ^b^
	*Caulerpa racemosa*	0.35	0.3 ± 0.1	0.5 ± 0.1	21.5 ± 2.1 ^b^
	*Codium isthmocladum*	0.65	0.1 ± 0.1	0.9 ± 0.3	10.5 ± 1.3 ^c^
	*Udotea flabellum*	0.76	0.3 ± 0.1	0.7 ± 0.2	40.1 ± 3.1 ^d^
Red seaweed	*Gracilaria caudata*	0.19	0.1 ± 0.1	0.8 ± 0.2	55.8 ± 1.8 ^e^
	*Gracilaria birdiae*	0.44	0.1 ± 0.1	0.7 ± 0.2	30.3 ± 2.1 ^a^

Data are expressed as mean ± standard deviation. Different letters indicate significant differences among samples according to one-way ANOVA followed by Tukey’s test (*p* < 0.05). TAC—total antioxidant capacity. * mg of ascorbic acid equivalent (AAE)/g of sample.

**Table 2:** Chemical composition of sulfated polysaccharide-rich extracts from *G. caudata*.

Sulfated Polysaccharides	Yield ^a^ (%)	Total Sugar (%)	Sulfate (%)	Protein (%)	Molar Ratio					
					Gal	Glu	Man	Xyl	Sulfate	MW (kDa)
F1.5	82.0	87.7	11.7	-	1.0	-	-	-	1.0	120 ± 03
F2.0	12.0	72.9	26.2	-	1.0	0.2	0.3	0.2	2.0	80 ± 04
F3.0	6.0	84.9	14.7	n.d	1.0	0.9	0.8	0.6	1.8	45 ± 02

^a^ All polysaccharides obtained were dried and weighed and total mass of each sample corresponded to 100%. Gal: galactose; Xyl: xylose; Man: mannose; Gluc: glucose; -Traces; n.d—not detected.

## Data Availability

Dataset generated during the current study are available from the corresponding author upon reasonable request.

## Abbreviations

The following abbreviations are used in this manuscript:

3T3-L1	Clonal line of preadipocytes derived from mouse embryonic fibroblasts
BMI	Body Mass Index
CHO	Chinese hamster ovary cells
DMEM	Dulbecco’s Modified Eagle Medium
EDTA	2,2′,2″,2‴-(Ethane-1,2-diyldinitrilo) tetra-acetic acid
FBS	Sterile fetal bovine serum
GSH	Reduced glutathione
H_2_DCF-DA	2′,7′-dichlorodihydrofluorescein diacetate
Hela	Human cervical adenocarcinoma cells
HPSEC	High-performance size-exclusion chromatography
MDA	Malondialdehyde
MTT	3-[4,5-dimethylthiazol-2-yl]-2,5- diphenyltetrazolium bromide
NBT	Nitro blue tetrazolium
NC	Negative control
OD	Optical density
PANC-1	Epithelioid carcinoma cells from human pancreatic duct
PBS	Phosphate-buffered saline
PC3	human prostate cancer cells
PDA	1,3-diaminopropane–acetate buffer
RAW 264.7	murine macrophage cells
ROS	reactive oxygen species
RPMI	Roswell Park Memorial Institute medium
SD	Standard deviation
SOD	Superoxide dismutase
SPREs	Sulfated polysaccharide-rich extracts
TAC	Total antioxidant capacity
TCA	Trichloroacetic acid
